# MHC2TA mRNA levels and human herpesvirus 6 in multiple sclerosis patients treated with interferon beta along two-year follow-up

**DOI:** 10.1186/1471-2377-12-107

**Published:** 2012-09-25

**Authors:** Maria Inmaculada Dominguez-Mozo, Marta Garcia-Montojo, Virginia De Las Heras, Angel Garcia-Martinez, Ana Maria Arias-Leal, Ignacio Casanova, Rafael Arroyo, Roberto Alvarez-Lafuente

**Affiliations:** 1 Servicio de Neurología. Hospital Clínico San Carlos, Instituto de Investigación Sanitaria del Hospital Clínico San Carlos (IdISSC), Madrid, Spain; 2 Laboratorio de Investigación de Esclerosis Múltiple (4a planta). Pabellón 8 - Hospital Clinico San Carlos, Ciudad Universitaria, 28040, Madrid, Spain

**Keywords:** HHV-6, MHC2TA, Multiple sclerosis, Quantitative RT-PCR, Interferon beta

## Abstract

**Background:**

In previous studies we found that MHC2TA +1614 genotype frequency was very different when MS patients with and without human herpesvirus 6 (HHV-6) in serum samples were compared; a different clinical behavior was also described. The purpose of the study was: 1. To evaluate if MHC2TA expression in MS patients was influenced by interferon beta (IFN-beta) treatment. 2. To study MHC2TA expression in MS patients with and without minor allele C. 3. To analyze the relation between MHC2TA mRNA levels and HHV-6 active infection in MS patients.

**Methods:**

Blood and serum samples of 154 MS patients were collected in five programmed visits: basal (prior to beginning IFN-beta treatment), six, twelve, eighteen and twenty-four months later. HHV-6 in serum and MHC2TA mRNA levels were evaluated by PCR and RT-PCR, respectively. Neutralizing antibodies (NAbs) against IFN-beta were analyzed by the cytopathic effect assay.

**Results:**

We found that MHC2TA mRNA levels were significantly lower among MS patients with HHV-6 active infection at the basal visit (without treatment) than in those MS patients without HHV-6 active infection at the basal visit (p = 0.012); in all the positive samples we only found variant A. Furthermore, 58/99 (58.6%) MS patients without HHV-6 along the five programmed visits and an increase of MHC2TA expression after two-years of IFN-beta treatment were clinical responders vs. 5/21 (23.8%) among those MS patients with HHV-6 and a decrease of MHC2TA mRNA levels along the two-years with IFN-beta treatment (p = 0.004); no differences were found between patients with and without NAbs.

**Conclusions:**

MHC2TA mRNA levels could be decreased by the active replication of HHV-6; the absence of HHV-6 in serum and the increase of MHC2TA expression could be further studied as markers of good clinical response to IFN-beta treatment.

## Background

In a previous study in the Spanish multiple sclerosis (MS) population, our group found that the MHC2TA +1614 genotype frequency was very different when MS patients with human herpesvirus 6 (HHV-6) were compared with MS patients without this virus [1]. The proportion of carriers of the minor allele (C) was higher in MS patients with HHV-6 than in patients without HHV-6, and then in controls. These results provided the evidence of an interaction between genetic and environmental factors that might lead to MS by an unknown mechanism. In a subsequent study [2], we verified the previous association that we had found between the HHV-6 active replication and the presence of MHC2TA rs4774C; furthermore, we found that those MS patients with minor allele C and HHV-6 active infection had different clinical behavior since they were worse clinical responders to IFN-beta treatment, and they had a higher progression in the first two years of the disease. Therefore, the presence of HHV-6 active replication and MHC2TA rs4774C could be possible markers of IFN-beta response.

In order to deepen this possible relationship we performed a new study with the following objectives: 1. To evaluate if MHC2TA expression in MS patients was influenced by interferon beta (IFN-beta) treatment. 2. To study MHC2TA expression in MS patients with and without minor allele C. 3. To analyze the relation between MHC2TA mRNA levels and HHV-6 active infection in MS patients.

## Methods

### Subjects

A total of 154 patients with clinically definite relapsing-remitting MS (RRMS) were included in the study (53 males, age ranging between 21-58 years, and 101 females, age ranging between 20-57 years). All patients were characterized as having RRMS for more than 2 years. All of them had been treated, at least, during two years, with interferon beta: IFN-beta-1a (Avonex, n = 15) 30 μg intramuscularly once weekly, IFN-beta-1b (Betaferon, n = 88) 8 MIU subcutaneously every other day, or IFN-beta-1a (Rebif, n = 51) 22 or 44 μg subcutaneously three times weekly, for more than two years. A control group of 154 healthy Spanish individuals was included for comparative purposes in the expression study. RRMS patients and controls were paired by age and sex; none of the healthy controls had relatives of first or second degree with MS or other autoimmune diseases, and none of them had received antiviral medication for at least 6 months before the enrolment in the study. The study conformed to the Helsinki Declaration and was approved by the local ethic committee (“Comité Ético de Investigación Clínica del Hospital Clínico San Carlos”), and all the participants received and signed the written informed consent before the enrolment.

### Collection of clinical data

The following clinical data were collected: number of relapses in the first two years of treatment, EDSS in the first two years of treatment, and response to IFN-beta treatment. We considered that clinical responders were those MS patients without EDSS progression and without relapses in the first two years of treatment with IFN-beta. Definition of progression was different depending on the pre-treatment EDSS score: 1) increase ≥ 1.5 points if pre-treatment EDSS = 0; 2) increase ≥ 1 point if pre-treatment EDSS ≥ 1 and ≤ 5; 3) increase ≥ 0.5 points if pre-treatment EDSS ≥ 5.5.

### Collection of samples

At the time of visit 10 ml of peripheral blood were drawn by vein puncture into sterile tubes with EDTA and directly used for DNA extraction, and 2 ml of serum were isolated in serum separator tubes by centrifugation and then were separate into aliquots and stored at –80°C. Among RRMS patients, two samples, blood and serum, were collected at the basal visit (before starting IFN-beta treatment), and a second pair of samples were obtained six (ranged 5-7), twelve (ranged 11-13), eighteen (ranged 16-20), and twenty-four months (ranged 22-26) after starting IFN-beta treatment.

### DNA extraction

Total DNA was isolated by DNA spin column technique of QIAamp DNA Blood Mini Kit (QIAGEN. Hilden. Germany), from 0.2 ml of blood, and QIAamp Ultrasens Virus Kit (QIAGEN), from 1 ml of serum, according to the manufacturer’s instructions. Each sample was extracted in duplicate. Two negative controls, consisting of reagents only, were processed with each set of eight samples.

### MHC2TA Genotyping

One polymorphism in the MHC2TA gene (rs4774) was analyzed by TaqMan Assay-on-Demand (C___381733_10) from Applied Bios stems, following manufacturer’s suggestions. This genetic marker conformed to Hardy-Weinberg equilibrium proportions in the control population (p > 0.05).

### Quantitative real-time PCR for HHV-6

PCR assays were performed in a Rotor-Gene 3000 (Corbett Research. Sydney. Australia). Primers and probes for detection of HHV-6 and PCR protocol are previously published [3].

### HHV-6 variant detection

HHV-6 variants (A and B), were characterized in the HHV-6 positive samples by quantitative PCR, as previously described [4].

### RNA extraction

Total RNA was extracted from peripheral blood using the QIAamp RNA Blood Mini Kit (QIAGEN. Hilden. Germany) following manufacturer instructions. Prior to RT, all the samples were digested with RNase-free DNase (Sigma, St. Louis. MO. USA), at 70°C for 15 min, to avoid any possible DNA contamination.

### Reverse transcription (RT)

RT was carried out with the Transcript or first strand cDNA synthesis kit (Roche Diagnostics, S.L. Barcelona. Spain). A 25°C incubation was followed by one cycle of 50°C for 1 hour and 1 cycle of 85°C for 5 minutes to inactivate the reaction and the cDNA was stored at –80°C until used for quantitative real-time PCR.

### Quantitative real-time PCR for cDNA of MHC2TA

To measure MHC2TA mRNA expression, quantitative real-time PCR was performed with a TaqMan® Gene Expression Assay ID Hs00172106_m1 from Applied Bios stems (Foster City. CA. USA), following the manufacturer instructions. As MHC2TA transcriptional expression was expressed in a relative manner, the results were normalized using the housekeeping gene beta-actin as reference to avoid differences due to possible RNA degradation/contamination or different reverse transcription efficiency (Beta-actin control kit. Eurogentec. Liege. Belgium), as it has been previously described [5]. Results were normalized to normalization standard samples of cDNA obtained from matched healthy controls, run at the same time as the cDNA samples of the experimental points; these controls were assigned the normalization ratio (NR) of 1.

### Cytopathic effect (CPE) assay

NAbs were measured through a CPE assay using the encephalomyocarditis (EMC) murine virus [6] on human lung carcinoma cell line (A549). Monolayers of A549 cells were prepared in 96 wells plates. Serum samples were inactivated at 56°C for 30 min. Then, they were diluted and incubated for 1 hour with IFN-beta 1b at a final concentration of 10 U/ml, in order to allow the joint of NAbs to IFN-beta. After that, the mixture was added to the plates with A549 cells monolayers and incubated overnight. Cells were then infected with EMC murine virus and viable cells were quantified 24 hours later by staining with crystal violet in 20% ethanol. Acetic acid 33% was added to the plates and absorbance at 620 nm was measured. The titres were calculated according to Kawade’s formula [7], and expressed in tenfold reduction unit (TRU). Samples were considered positives if titres were > 20.

### Statistical analysis

Odds ratios (O.R.) and exact 95 percent confidence intervals (C.I.) were calculated with standard microcomputer software: Epi Info v. 6.02 (CDC, Atlanta, USA) and SPSS Ver. 15.0 (SPSS Inc.). The chi-square test was used to compare qualitative variables. As MHC2TA expression levels (NR) were not normally distributed we used non- parametric tests for its analysis: Kruskal-Wallis method for comparison of more than two groups and U-Mann Whitney test to compare NRs between two groups. We considered statistically significant differences when p < 0.05.

## Results

The NAbs prevalence along the two years follow-up was 22.1% (34/154): 6.7% for MS patients treated with Avonex (1/15), 11.8% for MS patients treated with Rebif (6/51) and 30.7% among MS patients treated with Betaferon (27/88). At the six month visit the NAbs prevalence was 9.7% (15/154), 13.6% at the twelve month visit (21/154), 17.5% at 18-month visit (27/154) and 16.2% two years after the beginning of the treatment (25/154).

**Figure 1:**
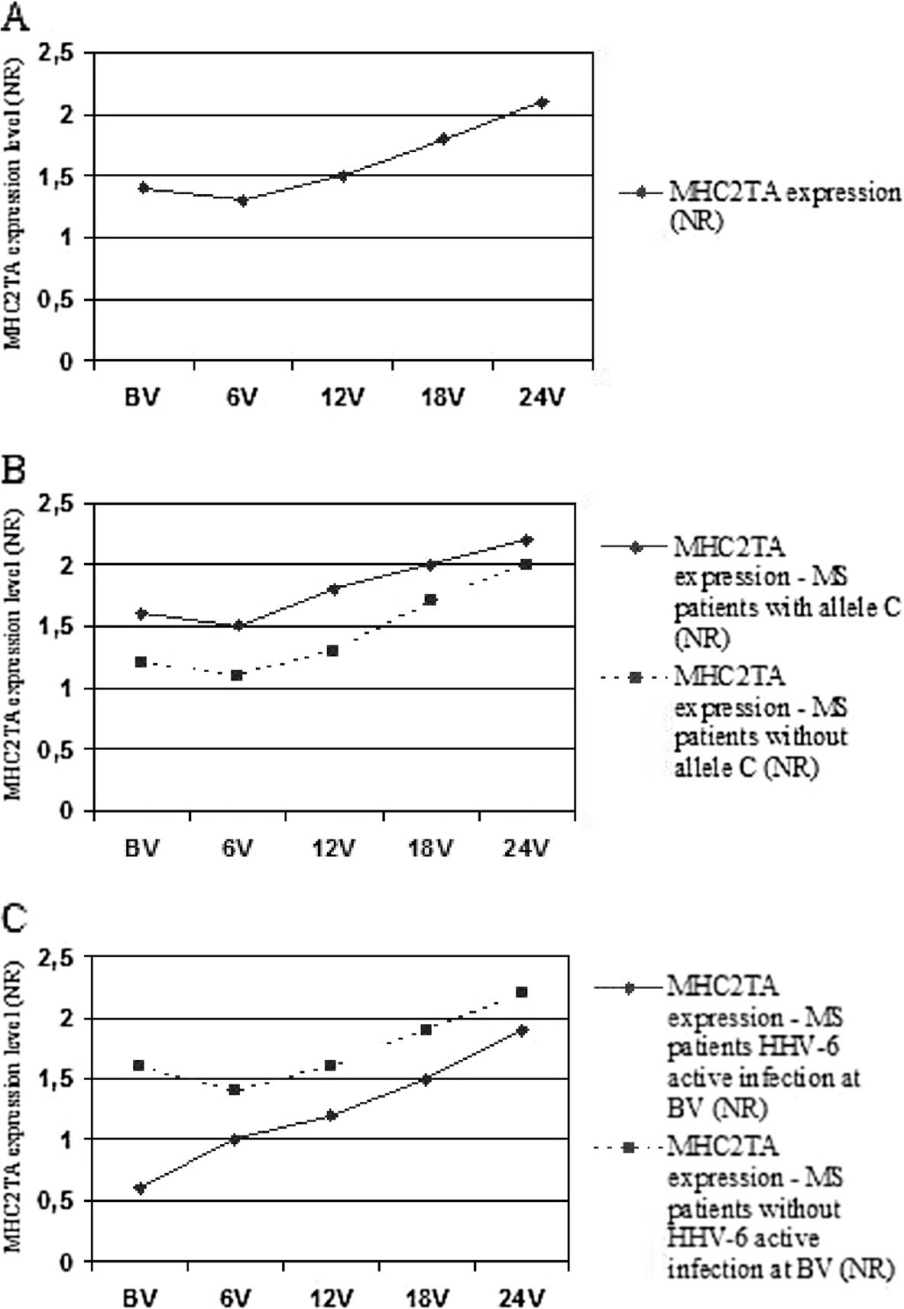
**MHC2TA expression levels as NR. A**. Mean NR at each visit. **B**. MS patients with minor allele C vs. MS patients without minor allele C. **C**. MS patients with minor allele C and HHV-6 in serum at basal visit vs. MS patients without minor allele C and without HHV-6 active infection at basal visit.

When we analyzed the influence of IFN-beta treatment on the expression of MHC2TA gene in the whole MS population, we did not find any statistical significant difference between the basal visit and the 24-month visit (p = 0.134): mean NR was 1.4 at basal visit, 1.3 after six months of treatment, 1.5 twelve months later, 1.8 at the 18-month visit, and 2.1 two years later after starting IFN-beta treatment (Figure 1A). No statistical significant differences were found between MS patients with and without NAbs.

When we studied MHC2TA expression in MS patients with and without minor allele C (82 MS patients were GG genotype, 64 were GC genotype, and 8 were CC genotype), we did not find any statistical significant difference for any of the scheduled visits: we found NRs of 1.6, 1.5, 1.8, 2, and 2.2 for the basal visit and 6, 12, 18 and 24 months later, respectively, among MS patients with minor allele C, and NRs of 1.2, 1.1, 1.3, 1.7, and 2 for the basal visit and 6, 12, 18 and 24 months later, respectively, among MS patients without minor allele C (Figure 1B); MS patients with allele C were normalized with healthy subjects with allele C, and MS patients with allele G were normalized with healthy subjects with allele G.

A total of 36 MS patients were positive for HHV-6 in serum at least once (28 were positive for the first time at the basal visit, 5 at the 6-month visit, 2 at the 12-month visit and 1 at the 18-month visit), while only 3/154 (1.9%) healthy subjects were positive for HHV-6 in their serum samples. When we analyzed the MHC2TA mRNA levels in MS patients with HHV-6 active infection at the basal visit (28/154; NR = 0.6, 1, 1.2, 1.5, 1.9, for the basal visit and 6, 12, 18 and 24 months later, respectively) and those without HHV-6 active infection at the basal visit (126/154; NR = 1.6, 1.4, 1.6, 1.9, 2.2, for the basal visit and 6, 12, 18 and 24 months later, respectively), we found a statistical significant difference for the NRs at the basal visit (p = 0.012), but not for the other scheduled visits, when MS patients were under IFN-beta treatment (Figure 1C); MS patients with HHV-6 active infection were normalized with healthy subjects with HHV-6 active infection, and MS patients without HHV-6 active infection were normalized with healthy subjects without HHV-6 active infection. No statistical significant differences were found in NAbs prevalence among MS patients with and without HHV-6 at basal visit. At table 1 we show the evolution of the HHV-6 DNA prevalence in the serum along the five scheduled visits. When we analyzed all the positive samples, we only found variant A.

**Table 1:** HHV-6 DNA prevalence along the five scheduled visits

	**VB**	**6M**	**12M**	**18M**	**24M**
**HHV-6 DNA prevalence in serum**	28 / 154	22 / 154	19 / 154	18 / 154	16 / 154
	18.2%	14.3%	12.3%	11.7%	10.4%
**p (between the basal visit and the 24-month visit)**					0.050
**OR (between the basal visit and the 24-month visit)**					1.92 (0.95-3.91)

Finally, when we analyzed the relationship between the increase/decrease of MHC2TA expression levels and the presence/absence of HHV-6 in serum with the clinical response to IFN-beta treatment (see Table 2), we found a statistical significant difference: only 5/21 (23.8%) MS patients with at least one HHV-6 positive serum sample among the five programmed visits and with a decrease of the MHC2TA mRNA levels after two years of IFN-beta treatment were clinical responders, vs. 58/99 (58.6%) MS patients without HHV-6 in the five programmed visits and with an increase of the MHC2TA level after two years of IFN-beta treatment (p = 0.004).

**Table 2:** Clinical response to IFN-beta treatment in MS patients with a decrease of MHC2TA mRNA levels and HHV-6 in serum (at least once among the five programmed visits) vs. MS patients with an increase of MHC2TA mRNA level and without HHV-6 in serum, after two years of IFN-beta treatment

	**IFN-beta clinical response**			EDSS*	Relapses**
	**N**	**Resp.**	**%**		
**MHC2TA mRNA level decreased + HHV-6 in serum**	21	5	23.8	1.5	1.2
**MHC2TA mRNA level increased without HHV-6 in serum**	99	58	58.6	- 0.5	0.4
**p**		0.004			

* Mean (EDSS at 24-month minus EDSS at basal visit).

** Mean of relapses after two years of IFN-beta treatment.

## Discussion

IFN-beta is one of the therapeutic options of relapsing remitting MS to date, but basic mechanisms underlying the beneficial effects of IFN-beta are still under investigation [8]. Interferons play essential roles in the front line of host defense against viral infections and in immunosurveillance for malignant cells. In general, it has been described that IFN-beta is a far less potent inducer of MHCII than IFN-gamma [9]. Our results show a light increase of the MHC2TA mRNA levels from the basal visit (prior starting IFN-beta treatment) to the 24-month visit (Figure 1A), but we did not find any statistical significant difference (p = 0.134). However, this was a heterogeneous population with different polymorphisms at the MHC2TA gene, with MS patients with and without viral infections along the two-year IFN-beta treatment, and with different clinical behavior (we found MS patients that were clinical responders to IFN-beta and other MS patients not). Therefore, we analyzed the contribution of each one of these factors to the observed changes in the MHC2TA mRNA levels.

As we have previously mentioned, all the MS patients included in the study were characterized for the exonic rs4774 polymorphism in the MHC2TA gene, and they were classified as carriers of the minor allele C or carriers of the allele G. As we can see in Figure 1B, we did not find any difference between these two MS populations. The amino acid change (from alanine to glycine) brought about by the rs4774C allele seems to be not related to a different expression pattern of the MHC2TA gene, but in this study we did not analyze the MHC2TA protein levels or the effectiveness of this protein as class II transactivator. Further studies should be performed to clarify this question.

MHC2TA plays a critical role in the control of antiviral immune response [10] and encodes the class II transactivator, MHC2TA, which is essential for class II transcription and expression [11]. As a master regulator of MHC class II genes, MHC2TA is an attractive target for modulation by pathogens that are controlled by CD4+ T cells [12]. Among such pathogens, Human Parainfluenza Virus Type 3 [13], or human cytomegalovirus (HCMV) (another human beta-herpesvirus such as HHV-6) are able to suppress induction of class II MHC expression that results in decreased levels of MHC2TA mRNA levels [14-16]. When we analyzed the effect of the HHV-6 infection over the expression of the MHC2TA gene, we found a statistical significant difference (p = 0.012) between the MHC2TA mRNA levels among those MS patients that were positive for this virus at the basal visit (lower expression level) and those that were negative (higher expression level). The differences were no longer statistically significant as IFN-beta treatment progressed. Although it should be further studied, HHV-6 seems to be able to reduce MHC2TA expression; then, the antiviral effect of IFN-beta treatment, that has been previously described, among others [17], by our group [18], leads to a decrease in the HHV-6 active replication rate, and an observed increasing of the MHC2TA mRNA levels under the progressive absence of HHV-6 in the serum along the scheduled visits (see Table 1). Finally, these last results were related to the clinical response to IFN-beta treatment (see Table 2): 58/99 (58.6%) MS patients with an increase of MHC2TA mRNA levels and without HHV-6 in the five programmed visits were free of progression an relapses under IFN-beta treatment, while only 5/21 (23.8%) of those MS patients with a decrease of MHC2TA expression and with HHV-6 in at least one of the five programmed visits could be consider as clinical responder.

## Conclusions

Then, we can conclude that MHC2TA expression seems to be lightly influenced by IFN-beta treatment (through the activation of an antiviral mechanism or cascade?). The rs4774 polymorphism in the MHC2TA gene does not seem to have any influence in the MHC2TA mRNA levels, but it could be further studied as a possible marker of clinical response to IFN-beta treatment. Finally, as previously described for other herpesvirus, like cytomegalovirus, MHC2TA mRNA levels could be also decreased by the active replication of HHV-6; the absence of HHV-6 in serum and the increase of MHC2TA expression could be further studied as markers of a good clinical response to IFN-beta treatment.

## Competing interests

The authors declare no competing interests.

## Authors’ contributions

RAL and RA conceived of the study, and participated in its design and coordination and helped to draft the manuscript. MID carried out the molecular studies, and drafted the manuscript. AMA and AG processed the samples and drafted the manuscript. MG and VD participated in the design of the study and performed the statistical analysis. IC analyzed the data and drafted the manuscript. All authors read and approved the final manuscript.

## Pre-publication history

The pre-publication history for this paper can be accessed here:

http://www.biomedcentral.com/1471-2377/12/107/prepub
